# MS, pregnancy and COVID-19

**DOI:** 10.1177/1352458520949152

**Published:** 2020-08-17

**Authors:** Charmaine Yam, Vilija Jokubaitis, Kerstin Hellwig, Ruth Dobson

**Affiliations:** Department of Neurology, Royal Free Hospital NHS Trust, London, UK; Department of Neuroscience, Monash University, Melbourne, VIC, Australia; Department of Neurology, St. Josef Hospital, Ruhr-University Bochum, Bochum, Germany; Preventive Neurology Unit, Wolfson Institute of Preventive Medicine, Queen Mary University of London, London, UK/Department of Neurology, Royal London Hospital, Barts Health NHS Trust, London, UK

**Keywords:** Multiple sclerosis, COVID-19, SARS-COV-2, pregnancy, antenatal care

## Abstract

Concerns regarding infection with the novel coronavirus SARS-CoV-2 leading to COVID-19 are particularly marked for pregnant women with autoimmune diseases such as multiple sclerosis (MS). There is currently a relative paucity of information to guide advice given to and the clinical management of these individuals. Much of the limited available data around COVID-19 and pregnancy derives from the obstetric literature, and as such, neurologists may not be familiar with the general principles underlying current advice. In this article, we discuss the impact of potential infection on the pregnant woman, the impact on her baby, the impact of the current pandemic on antenatal care, and the interaction between COVID-19, MS and pregnancy. This review provides a framework for neurologists to use to guide the individualised advice given to both pregnant women with MS, and those women with MS who are considering pregnancy. This includes evidence derived from previous novel coronavirus infections, and emerging evidence from the current pandemic.

## Background

Concerns regarding infection with the novel coronavirus SARS-CoV-2 leading to COVID-19 are particularly marked for both people with multiple sclerosis (MS) and pregnant women. There is a relative paucity of information to guide the advice given to, and clinical management of, these individuals. Both individuals with MS and pregnant women are identified as ‘vulnerable’, and stringent social distancing is recommended; however, the precise advice given to people with MS varies. Early data from Italy suggest that there may not be a significantly enhanced risk of adverse outcomes in people with MS;^1^ however these data remain preliminary, and the impact of immunosuppression remains to be determined. There is increasing evidence that certain co-morbidities, such as hypertension, are associated with increased severity of COVID-19^2^. Recent data originating from Italian cohorts, currently available in preprint form only, has suggested that exposure to anti-B cell therapies such as Ocrelizumab and/or recent steroid exposure may confer increased risk of severe COVID-19. Much of the limited available data around COVID-19 and pregnancy derive from the obstetric literature, and as such, neurologists may not be familiar with the general principles underlying current advice.

Responses to viral infections differ in pregnant women, and pregnant women are in general at increased risk of respiratory infection. Some infections, such as influenza may be more severe.^3^ This is due to direct changes in the immune cell repertoire, as well as physical changes such as elevation of the diaphragm and splaying of the thoracic cage due to the enlarged uterus. These physical changes decrease the functional residual capacity and ability to clear secretions, in the presence of increased oxygen consumption during pregnancy.^4^

Previous novel coronavirus infections (SARS, MERS) have been associated with increased risk of adverse outcomes including spontaneous miscarriage with infection in the first trimester of pregnancy and preterm birth and intrauterine growth restriction (IUGR) with infection in the third trimester.^5,6^ Case fatality rates in pregnant women were estimated to be up to 25% in SARS-CoV^5^ and 27% in MERS-CoV.^7^ Fortunately, this pattern has not been replicated thus far in SARS-CoV-2/COVID-19.^8^ In contrast to SARS and MERS, there appears to be a cohort of minimally symptomatic or even asymptomatic individuals who carry COVID-19, some of whom may be pregnant women.^9,10^ However, due to the fact that COVID-19 pneumonia can cause rapidly progressive diffuse bilateral interstitial lung disease, there is a potentially high risk of respiratory decompensation and hypoxaemic respiratory failure in symptomatic pregnant women,^11^ which likely contributes to the emerging increased risk of critical care admission in pregnant women with COVID-19.^12^

There are, therefore, a number of key clinical considerations regarding COVID-19 and MS in pregnancy. Broadly speaking, these are (1) the impact of potential infection on the pregnant woman, (2) the impact on her baby, (3) the impact of the current pandemic on antenatal care, and (4) the interaction between COVID-19, MS and pregnancy. In general, women with MS who are also pregnant should be advised to follow appropriate social distancing measures and/or shielding measures depending on their immunosuppressant exposure and additional clinical co-morbidities. It is important that pregnant women are advised to maintain contact with obstetric services as appropriate to their particular circumstances, as there are mounting concerns regarding poor outcomes related to lack of obstetric input. At present, this is a rapidly evolving field, and guidelines are frequently updated. The number of currently pregnant women with MS is relatively small, and so clinical experience with this group is relatively limited, but increasing.

## COVID-19 in pregnancy

### Susceptibility to COVID-19 during pregnancy – maternal outcomes

Transmission of COVID-19 is mainly via small respiratory droplets (sneezing, coughing or interacting with someone less than 1 m away) or via surfaces and touching the nose, mouth or eyes. It enters respiratory epithelium cells via the ACE-2 receptor, which is expressed throughout the upper and lower respiratory tract, and has been associated with a variety of clinical presentations related to upper and lower respiratory tract infection.

The innate and adaptive immune systems undergo various fluid adaptations throughout pregnancy that are precisely timed to promote pregnancy success.^13^ These include localised pro-inflammatory environments during implantation, tolerogenic adaptations during foetal growth and development in the first and second trimester, followed by a second pro-inflammatory phase in preparation for parturition in the third trimester.^14^ The preferential Th2 immune response in the first and second trimesters leaves the mother more vulnerable to viral infections, which are usually contained by the Th1 immune system.^15^ The impact of critical illness during pregnancy on maternal and/or foetal outcomes is not insignificant, regardless of underlying aetiology. Physiological changes during pregnancy place additional strain on the cardiopulmonary system of the mother, including increased metabolic, and hence oxygen demands, which may be exacerbated by anaemia. In addition, diaphragmatic splinting by the gravid uterus reduces total lung volume and effective oxygen transfer and impacts the ability to clear secretions. Protracted respiratory compromise and maternal hypoxia drive the release of potent vasoconstrictors including endothelin-1 and hypoxia-inducible factor, which can lead to placental hypoperfusion, increasing the risk of foetal growth restriction.^16^ Mothers with pneumonia are more likely to deliver before 34 weeks of gestation, which may be secondary to prostaglandin production or the host’s inflammatory response to infection.^4^

Several case series (Table 1) and systematic reviews (Table 2) have been published, showing maternal and neonatal outcomes following maternal COVID-19 infection. Higher rates of critical illness have been reported in MERS and SARS with 41% and 35% of affected pregnant women requiring mechanical ventilation, respectively, and maternal mortality of 25% and 18%, respectively.^8^ In contrast, while the majority of pregnant women with COVID-19 had radiological evidence of pneumonia (X-ray or computed tomography (CT)), there have not been significantly increased maternal deaths compared to non-pregnant individuals with COVID-19,^17^ although emerging evidence suggests an increased risk of critical care admission.^12^

**Table 1.** Existing case series containing >20 pregnant women with COVID-19 in pregnancy.

Study	Study design	Country	Number of COVID19 + pregnant women	Number of neonates delivered	Maternal outcomes	Neonatal/foetal outcomes
Chen et al.^18^	Case series	China	118	68	Mild disease (92%) Hypoxaemia (8%) Non-invasive ventilation (0.8%) Caesarean section (61%)	Preterm birth (21%) Spontaneous abortion (2.5%) Neonatal hypoxia (0%)
Yan et al.^19^	Case series	China	116	100	Symptomatic disease (76.7%) Severe disease (6.9%) Maternal death (0%)	Spontaneous abortion (0.9%) Preterm birth (21.2%) Neonatal death (1%) Neonatal COVID-19 infection (0%; *n* = 86)
Savasi et al.^20^	Case series	Italy	77	57	Symptomatic disease (84%) Severe disease (18%) ICU admission (8%) ECMO (1.3%) Maternal death (0%)	Preterm delivery (21%) NICU admission (16%) Neonatal COVID-19 infection (7%)
London et al.^21^	Retrospective cohort study	USA	68 (65 in third trimester)	55	Symptomatic disease (67.6%) Respiratory support required (18%) Caesarean section (40%)	Preterm delivery (16.3%) Neonatal COVID-19 infection (0%)
Pereira et al.^22^	Case series	Spain	60	23	Maternal deaths (0%) Caesarean section (22%) Pneumonia (37.8%) Critically unwell (10%) ICU admission (2%)	Neonatal COVID-19 infection (PCR) (0%)
Lokken et al.^23^	Case series	USA	46	8	Symptomatic disease (93.5%) Required hospitalisation (16%) Severe disease (15%)	Stillbirth (unclear aetiology) (12.5%, *n* = 1) Preterm delivery at 33/40 (12.5%, *n* = 1)
Ferrazi et al.^24^	Case series	Italy	42	42	Pneumonia (45.2%) Required oxygen support (16.6%) Critical care admission (9.5%) Caesarean section (42.9%)	Preterm birth (26.2%) NICU admission (7.1%) Neonatal COVID-19 infection (7.1%)
Campbell et al.^9^	Case series	USA	30	30	Symptomatic disease (26.7%) Caesarean section (33.3%)	Preterm delivery (0%) Apgar score < 7 at 1 and 5 minutes (0%)
Yang et al.^25^	Case series	China	27	24 (1 set of twins)	Symptomatic disease (46.2%) Caesarean section (78%) Maternal death (0%)	Preterm delivery (4.3%) Low birth weight (8.3%) Neonatal COVID-19 infection (0%) Severe neonatal hypoxia (4.2%)

NICU: neonatal intensive care unit; PCR: polymerase chain reaction; ECMO: extracorporeal membrane oxygenation.

**Table 2.** Existing systematic reviews containing >100 pregnant women with COVID-19 in pregnancy.

Study	Study design	Country	Number of COVID-19 + pregnant women	Number of neonates delivered	Maternal outcomes	Neonatal/foetal outcomes
Elshafeey et al.^26^	Systematic review (33 studies)	China, Australia, Honduras, Iran, South Korea, Sweden, Turkey, USA, Netherlands	385	256	Symptoms: mild (95.6%), severe (3.6%), critical (0.8%) ICU admission (4.4%) Mechanical ventilation (1.6%) Maternal deaths (0.3%)	Neonatal COVID-19 infection (1.6%) Stillbirths (0.8%) Neonatal death (0.4%) Low birthweight (7.8%) Intrauterine foetal distress (7.8%) NICU admission (3.1%) Neonatal mechanical ventilation (1.2%)
Juan et al.^27^	Systematic review	China, Australia, Canada, France, Korea, Iran, Italy, Peru, Spain, Sweden, Turkey, USA	295 from case series 20 from case reports	219 (case series) 19 (case reports)	Case series: Pneumonia (0%–14%) Maternal deaths (2.4%) Case reports: Maternal deaths (10%)	Case series: Spontaneous miscarriage (1.4%) Foetal death/stillbirth (1.4%) Low birth weight (2.7%) Neonatal death (0.5%) Neonatal COVID-19 infection (1.9%) Case reports: Neonatal death (5.3%) Neonatal COVID-19 infection (10.5%)
Gajbhiye et al.^28^	Systematic review (case reports and series)	China (20 studies), Korea (1), USA (1), Honduras (1)	172	160	Pneumonia (100%) Preterm labour (21%) Premature rupture of membranes (8%) ICU admission (3%) ECMO (0.6%) Maternal deaths (0%)	Preterm birth (23%) Neonatal respiratory distress syndrome (14%) Neonatal pneumonia (14%) Low birth weight (11%) Small for gestational age (3%) Neonatal COVID-19 infection (11%) Stillbirth (1%) Neonatal death (1%)
Yang et al.^29^	Systematic review (18 studies)	-	114	84	Caesarean section (91%) Severe or critical illness (5.3%) ECMO (0.9%)	Stillbirth (1.2%) Neonatal death (1.2%) Preterm birth (21.3%) Low birth weight (5.3%) Neonatal COVID-19 infection (2.4%)
Zaigham and Andersson^30^	Systematic review	Sweden, USA, Korea, Honduras	108	75	Caesarean section (92%) Maternal ICU admission (3%) Maternal mortality (0%)	Intrauterine foetal death (1%) Neonatal mortality (1%) Neonatal COVID-19 infection (1%) Vertical transmission (1%)

NICU: neonatal intensive care unit; PCR: polymerase chain reaction; ECMO: extracorporeal membrane oxygenation.

### Immunological considerations related to COVID-19 in pregnancy

It has been speculated that the apparent differences in pregnancy-associated severity and mortality between COVID-19 and SARS/MERS arises from the fact that in SARS and MERS, there is a preferential activation of Th1 immunity, leading to a marked pro-inflammatory cytokine storm (interferon (IFN)-gamma, interleukin (IL)-1beta, IL-6 and IL-12) resulting in severe lung damage. In COVID-19, the inflammatory response appears different, with activation of both Th1 and Th2 immunity and preferential expression of IL-4 and IL-10. Hence, the inflammatory phase in COVID-19 is of lesser severity compared to SARS and MERS, and this response is even lower in pregnant women where there is already a predominant Th2 response.^8,31^

## Pregnancy and neonatal outcomes following COVID-19 infection

There has been a high rate of preterm births associated with COVID-19 (Tables 1 and 2). Reported rates of preterm birth have ranged between 18% and 43%, and neonatal pneumonia has been documented in up to 30%, although a systematic review estimated a lower rate of 8%,^28^ and many cases of neonatal respiratory infection were reported as “not due to COVID-19.”^8,28^ The rate of preterm births is potentially higher than previously documented in MERS and SARS-CoV (27% and 25%, respectively), while IUGR rates are comparable (9% vs 9% and 13% in MERS and SARS-CoV, respectively).^9^ A significant proportion of the preterm births reported in case series appear to be performed in order to protect maternal respiratory function. There were few miscarriages, stillbirths or neonatal deaths (2% or less; Tables 1 and 2).

Preterm birth and IUGR are associated with long-term sequelae in the child, including increased risk of chronic kidney disease, hypertension, cardiac remodelling due to increased peripheral vascular resistance and vascular growth, and reduced insulin sensitivity leading to higher risk of metabolic syndromes.^32^ There are also concerns regarding potential future risks of childhood inattention disorders secondary to protracted fever (two or more exposures) in the first or second trimesters mediated by hyperthermic injury to developing foetal neurons.^33^

Pathological studies of placental tissue from women with proven SARS-CoV2 infection have demonstrated an increased rate of features of maternal vascular malperfusion (MVM), including atherosis and fibrinoid necrosis and mural hypertrophy of membrane arterioles.^34^ MVM has been associated with oligohydramnios, foetal growth restriction, preterm birth and stillbirth in other studies, thus highlighting a potential mechanism of SARS-CoV-2 infection on pregnancy outcomes. There was an additional significant increase in the number of intervillous thrombi in placentas from women with COVID-19, which are likely to reflect the overall hypercoagulable state associated with infection.^34^ None of the placentas in this study were tested for SARS-CoV-2 viral RNA or protein. The ACE2 receptor is a co-receptor for viral entry into the host cell, which is thought to increase susceptibility to SARS-CoV-2 when upregulated.^35^ In pregnant women, there is evidence of increased ACE2 mRNA in the kidney, placenta and uterus,^36^ but further studies regarding the significance of this are needed.

There are increasing reports of COVID-19 infected neonates, raising the possibility of vertical transmission. In a retrospective review of nine mothers with COVID-19 who had a caesarean section in their third trimester, the amniotic fluid, cord blood, neonatal throat swabs and breast milk after first lactation were all negative for the virus, arguing against the notion of vertical transmission.^37^ In a pre-print systematic review, 313 neonates who had been tested for SARS-CoV-2 infection following maternal COVID-19 were identified from across the literature. Of these, 24 (8%) were reported to have evidence of SARS-CoV-2 infection, 7% using reverse transcription polymerase chain reaction (RT-PCR) and 3% antibody. When only those tested in the first 48 hours of life using either RT-PCR or IgM were included, 21/261 (8%) met the criteria for potential vertical transmission.^28^ There is, as yet, no evidence that neonates who are infected suffer COVID-19 pneumonia have poor outcomes but factors such as prematurity, asphyxia and bacterial sepsis contribute more in critically ill cases.^38^

## Antenatal and perinatal care

### Antenatal care

Antenatal care is essential to ensure the best pregnancy outcomes. However, regular attendance at hospital or outpatient settings potentially places obstetric patients at additional risk of contracting SARS-CoV-2. The reported prevalence of SARS-COV-2 in asymptomatic obstetric patients screened using nasopharyngeal swab PCR on admission to hospital during the height of the pandemic was 13.7%.^10^ Of all swab positive obstetric patients, 87.9% were asymptomatic. Only 10% of these initially asymptomatic patients developed fever prior to postpartum discharge (median length of stay 2 days). Further data from Connecticut reports a prevalence of SARS-CoV of 3.9% (30/770) among women presenting for childbirth in April 2020, of whom 22 (73.3%) were asymptomatic.^9^

Given the dual concerns of high rates of asymptomatically infected women, and the potential impact of lack of antenatal care, ‘virtual’ consultations should be carried out where appropriate. It is important to note that these are not possible or appropriate for all antenatal visits, and may unacceptably compromise care for some women. Where this is the case, for example, where physical examination and/or screening is required, women must be seen in person. Ensuring women are seen in one-stop clinics that cover all obstetric and medical needs in the same visit are needed to reduce potential exposure to the virus. For women with MS, this means coordinating obstetric and MS clinic reviews and blood tests to minimise hospital attendance and travel. Equipment such as ultrasound machines should be decontaminated after each use. Assessments that can be performed at home, such as blood pressure monitoring should be encouraged. There is increasing evidence that pregnant women of Black, Asian and Minority Ethnic backgrounds are at particular risk of requiring critical care for severe COVID-19;^39^ these women are also at increased risk of adverse pregnancy outcomes, particularly when they have other vulnerabilities such as lower socioeconomic status.^39,40^ It is vital to consider the potential impacts of these risks when considering care pathways.

The Royal College of Obstetricians and Gynaecologists sets out recommendations for managing pregnant women with suspected or confirmed symptomatic COVID-19.^41^ Recommendations include that appointments should be conducted remotely via telephone or videoconferencing where practical. Non-urgent routine appointments, including growth scans, antenatal community or secondary care appointments, should be delayed in symptomatic women until after the recommended period of self-isolation (7 days, or until fever settles) if safe to do so. Appointments should not be repeatedly rescheduled or delayed by more than 3 weeks. Women who need to be seen should always be seen without undue delay, and appropriate personal protective equipment (PPE) may be needed to facilitate this. In the rare cases of an obstetric emergency, obstetric management should be dealt as a matter of priority.

### Delivery options

Since there is no clear evidence of vertical transmission of SARS-CoV-2, there are no specific contraindications to vaginal delivery in the context of maternal infection, unless there is maternal deterioration or foetal compromise. Induction of labour should be minimised for indications that are not strictly necessary in order to minimise hospital inpatient stays.^41^ Furthermore, a case series showed no evidence of COVID-19 in vaginal secretion specimens, suggesting transmission to the newborn through mucosal contact during vaginal delivery is unlikely.^42^

Symptomatic COVID-19 does not warrant early delivery as a routine consideration. However, if mothers have severe COVID-19, requiring or anticipated to require cardiopulmonary support, early delivery should be considered to ensure maternal safety. The threshold for caesarean section may be lowered, especially if there is a delay in the first stage of labour, in order to reduce maternal inpatient stay, reduce maternal physical exertion during delivery and reduce exposure to the virus and the chance of cross-infection.^43^ This recommendation is not universal, however, with some recommending vaginal delivery via induction with eventual instrumental delivery if complicated or prolonged labour, to avoid unnecessary surgical complications in an already sick patient. They recommend emergency caesarean section only if there is septic shock, acute organ failure or foetal distress.^44^ The use of birthing pools in hospitals is discouraged due to the risk of infection via faeces.^45^ Caesarean sections should be carried out with consideration for the potential need for respiratory precautions and full PPE based on clinical risk, given the potential need for aerosol-generating procedures intraoperatively.

### Breastfeeding and post-partum considerations

#### General concerns around breastfeeding

The risks of potential transmission of the virus associated with breastfeeding should be balanced against the benefits for the individual mother–baby dyad. In general, women who give birth during the COVID-19 pandemic should be encouraged to breastfeed where they wish to do so, with appropriate precautions in case of asymptomatic infection. Potential benefits of breastfeeding include protection against allergies and subsequent autoimmune diseases, and maternal IgA secreted via breastmilk, which offers protection against respiratory and gastrointestinal infections. Some of these positive effects may also be long-lasting.^46^ The probable beneficial effect of breastfeeding on relapse rate in at least some women with MS is also a consideration in postpartum MS patients.^47–49^

In the context of clinical infection, SARS-COV-2 viral RNA has been detected in the breastmilk of at least one infected mother,^50^ although most prior studies have not detected viral particles.^18^ Transmission of infection through breast milk is well documented for cytomegalovirus (CMV), human immunodeficiency virus (HIV1) and human T-cell lymphotropic virus type 1 (HTLV-1) infections. It is thought that recurrent exposures to small amounts of virus in human milk over the breastfeeding period contribute to the high rate of transmission.^51^ In these cases, whether breastfeeding is contraindicated depends on the rate of transmission, potential complications of infection in the infant or neonate and whether the setting is in low or middle-income nations, where the risk of malnutrition and infectious disease may outweigh the risk of acquiring the maternal infection.

A significant concern is potential transmission of the virus via droplets from the respiratory tract of the mother during breastfeeding due to close contact. Current CDC guidelines for women with confirmed COVID-19 advise wearing a cloth face covering while breastfeeding and washing hands prior to each feed.^52^ Breast milk of SARS-CoV-2 positive women may be expressed as long as a dedicated breast pump is used with careful hand hygiene exercised and facemask used during milk expression. It is postulated that anti-SARS-COV-2 antibodies may be transferred to the infant via breastmilk, in a similar manner to other viral infections, but this has not been confirmed.

## COVID-19 impact on MS in pregnancy

Symptomatic viral infections have previously been associated with both a transient worsening of MS-related neurological symptoms (pseudo-relapses) and clinically defined relapses. While the MS relapse rate falls significantly during normal pregnancy, it does not completely abate, particularly in those women with more active MS who have received more aggressive disease-modifying treatments prior to pregnancy and then paused treatment during pregnancy.^53^ There is currently insufficient data to confirm or refute any association between COVID-19 infection and increased MS relapse rate either during pregnancy or in the post-partum ‘rebound’ period (or at any other time); however, there is no reason to suspect that there are any differences to other respiratory tract infections in this particular regard.

There is currently limited evidence surrounding the risk(s) associated with COVID-19 while on disease-modifying therapy (DMT). In the context of pregnancy, decisions regarding withholding or continuing DMTs should have already been discussed prior to or shortly after conception. Depending on disease-activity prior to conception, pregnant women are likely to either not be on treatment, or to be continuing treatment with a DMT that has been deemed as having a reasonable safety profile in pregnancy: usually interferon-beta, glatiramer acetate or natalizumab.^54^ These DMTs are not directly immunosuppressive and therefore are currently not routinely being stopped in response to the COVID-19 pandemic.^55^

Induction, ‘lymphodepleting’ DMTs, which may be used some months prior to pregnancy to induce disease remission prior to trying to conceive, includes ocrelizumab, alemtuzumab, rituximab and cladribine. Caution is currently recommended when initiating these treatments; however, advice is changing as evidence increases;^55^ the most recent evidence, available in preprint form only, indicates that there may be potentially increased risk of severe COVID-19 in those treated with anti-B cell monoclonal antibodies.Treatment decisions should be individualised according to disease activity and relapse risk, the number of cycles already taken and co-morbidities associated with potential complications following COVID-19 infection, such as smoking, immobility and increased age.^55^ While relapses are less common during pregnancy, if they do occur, corticosteroid treatment may be considered as per below.

### Treatment of relapses

MS relapses are known to be more common in the post-partum period, and the use of high-dose corticosteroids to treat these may lead to concerns regarding increased susceptibility to infection. The use of high-dose corticosteroids can suppress antibody responses, with potential implications for both the breastfeeding mother and passive immunity in the newborn. There is limited data on the impact of corticosteroids on the severity or disease course of COVID-19; most advice is based on data from the SARS-CoV epidemic in 2002–2004. Studies in humans and mice demonstrate that corticosteroids appear effective in reducing immunopathological damage, mainly through reducing Th1 immune responses,^56–59^ but concerns remain surrounding the promotion of viral rebound, enhancing viral replication and association with adverse effects, such as acute respiratory distress syndrome, particularly in the context of longer steroid courses.^60,61^ One randomised controlled trial examining SARS-CoV measured viral load at regular intervals in non-intubated cases and found higher concentrations of viral RNA in week 2–3 of infection in those treated with early corticosteroids (<7 days of illness) compared with placebo.^60^

Recently, a systemic review and meta-analysis of 5270 coronavirus-infected patients from 15 articles (11 including SARS-CoV infection, 2 including MERS-CoV infection and 2 including SARS-CoV-2 infection) found that corticosteroids were likely to be used in patients with critical illness, were associated with a higher mortality rate (relative risk (RR) = 2.11, 95% confidence interval (CI): 1.13–3.94) and longer length of inpatient stay and higher rate of bacterial infection (RR = 2.08, 95% CI: 1.54–2.81); however, selection bias and other confounding factors may have played a role.^62^ While the RECOVERY trial demonstrated a significant benefit of dexamethasone in those requiring supplemental oxygen,^63^ Italian data, available in preprint form only at present, suggests that the risk of severe COVID-19 is increased in those who have recently received high-dose steroids in the context of MS relapse. In view of this, for MS patients, corticosteroid-induced lymphopenia and immunosuppression are important considerations, especially when methylprednisolone dosing for MS relapses is frequently high; up to 500 mg to 1 g daily, albeit usually short courses of 3–5 days. The risks of steroid pulsing in post-partum MS relapses, where DMT options are limited, will need to be balanced against current and potential disability.

## Registries for pregnant women with COVID, alongside MS COVID registries

MS registries around have rapidly adapted to collect data on COVID-19 in MS. These include the COViMS US Registry MS ReCOV, MuSC-19 Italian registry, Swedish Registry, MSBase, Spanish MS Covid-19 Registry, French Registry, OPTIMISE-MS, UK MS Register COVID-19 sub-study, German MS Register, The Australian MS Longitudinal Study, ABEM-Brazilian MS Patients Association, Cleveland Clinic Registry and Esclerosis Multiple Argentina (EMA) COVID-19 survey. The Global Data Sharing Initiative and currently collating COVID-19 data from many of these. In addition, the UK Obstetric Surveillance System (UKOSS) launched a registry for all women admitted to UK hospitals with confirmed COVID-19 in pregnancy to assess the outcomes for mother and the infant. Given the relatively small number of pregnant MS women at any one time, it is vital that they are included in all such registry efforts as a subgroup of interest.

### Recommendations and conclusions

Current data appear to indicate an increased risk of preterm birth in pregnant women with COVID-19. Pregnant women do not appear to be at greater risk of COVID-19 infection; while the risk of critical care appears increased relative to the general population, there does not appear to be a significantly higher mortality rate. Management of pregnant women with MS comes with additional considerations due to the unique immunological environment during pregnancy and postpartum, immunomodulatory therapy use and implications for disease activity. Integrating care where possible to minimise hospital visits is an important way by which care can be delivered as safely as possible.

Current advice is to exercise stringent social distancing in pregnant women who are greater than 28 weeks of gestation, and this advice should be followed for pregnant women with MS. However, the wider implications of isolation and/or shielding must not be overlooked, particularly in those with chronic diseases such as MS – there is increasing concern regarding the long-term impact on mental and physical health. Consideration must be made to how services can respond to the needs of this small, but highly vulnerable, group, and neurologists should be aware of how service design can positively or negatively impact on their patients. Neurologists may be approached by patients who are either pregnant, or considering pregnancy, and an awareness of risks and areas of relative reassurance is needed, in order to enable patients to make the best decisions informed by current evidence.
